# Comparative analysis of authorship trends in the Journal of Hand Surgery European and American volumes: A bibliometric analysis

**DOI:** 10.1016/j.amsu.2020.05.015

**Published:** 2020-05-24

**Authors:** Alexander W. Peters, Michael K. Savaglio, Zachary J. Gunderson, Gremah Adam, Anthony J. Milto, Elizabeth C. Whipple, Randall T. Loder, Melissa A. Kacena

**Affiliations:** a Department of Orthopaedic Surgery, Indiana University School of Medicine, Indianapolis, IN, USA; b Ruth Lilly Medical Library, Indiana University School of Medicine, Indianapolis, IN, USA

**Keywords:** Bibliometric, Authorship trends, Gender, Comparative analysis

## Abstract

**Background:**

The purpose of this study was to better understand the authorship publishing trends in the field of hand surgery. To accomplish this, a comparative analysis was completed between the European and American volumes of the *Journal of Hand Surgery (JHSE* and *JHSA)* over the past three decades. Well-established bibliometric methods were used to examine one representative year from each of the past three decades. The focus of the study was to examine changes in author gender over time as well as to compare authorship trends across the two volumes.

**Materials and methods:**

All *JHSA* and *JHSE* publications from 1985, 1995, 2005, and 2015 were placed into a Microsoft Excel spreadsheet. Data was collected for each publication including the gender of first and corresponding authors, corresponding author position, corresponding author country of origin, number of credited institutions, authors, printed pages, and references. Countries were grouped by regions.

**Results:**

A total of 450 and 763 manuscripts from *JHSE* and *JHSA*, respectively, met inclusion criteria. *JHSE* and *JHSA* both showed increases in most variables analyzed over time. Both journals showed an increase in female first and corresponding authors. *JHSE* and *JHSA* displayed a rise in collaboration between institutions and countries.

**Conclusions:**

Both *JHSE* and *JHSA* display increasing female inclusion in the hand surgery literature, which has traditionally been a male dominated field. The observed increase in collaboration between institutions and countries is likely linked to advances in technology that allow sharing of information more conveniently and reliably than was previously possible. As further advances are made socially and technologically, hopefully these trends will continue, leading to faster and higher quality research being generated in the field of hand surgery.

## Highlights

• Orthopaedic hand literature has seen increases in collaboration over the past 30 years.
• Significant increase in female first authors in the orthopaedic hand literature.
• Female first and corresponding author manuscripts received the most citations.

## 1 Introduction

The *Journal of Hand Surgery®* (*JHS*) is a monthly, peer-reviewed journal that publishes original manuscripts concerning the diagnosis, treatment, and pathophysiology of diseases and conditions of the upper extremity and hand. There are American (*JHSA*) and European (*JHSE*) volumes. Due to the considerable influence of these journals in the field of hand surgery, we performed a comparative analysis on the bibliometric trends in authorship and collaboration over the past 30 years between *JHSA* and *JHSE*. This analysis allowed for the comparison of bibliometric trends across two continents and provided insight about the differences in authorship and collaboration trends between these volumes of *JHS*.

This study confirms and expands upon a 2016 paper by Mei et al. in the *International Journal of Surgery*, which found that there was a significant increase in hand and wrist research published between 2005 and 2014 [1]. Mei et al. examined regional differences in publishing activity and included data from *JHSA* and *JHSE* as well as *Journal of Hand Therapy* and *Hand Clinics.* Furthermore, this study expands upon a recent bibliometric analysis in *JHSA* [2]. Bibliometric variables in this study included the following: author gender, mentorship relationships between first and corresponding authors, and geographic diversity.

Medicine has been a male dominated field in the past. Recently, the gender gap in medicine has been closing. Women currently account for 47% and 33% of active physicians in the United Kingdom and the United States, respectively [3,4], and in the United States women comprise 47% of United States medical school matriculants [5,6]. However, there are gender differences between the various surgical specialties and also within academic medicine [7]. In 2006, 28% of general surgeons and 12% of orthopaedic surgeons in England were women [8]. Of all orthopaedic surgery subspecialties in the United States, pediatric orthopaedics has the highest percentage of women entering at 25% and spine the lowest at 3%, with the data for hand not given [9]. In 2016, women accounted for 19% of general surgeons, and 5% of orthopaedic surgeons in the United States [10]. Women comprise 30% of clinical faculty positions, but only 15% of positions in surgical specialties [4,6,9]. There is increasing emphasis on manuscript publication in obtaining registrar/residency and fellowship positions, as well as promotion and the ability to secure grant funding in academia [9,11,12]. Understanding the effects of gender differences in authorship may assist in improving gender equality in the field of hand surgery and assist mentors in counseling mentees, whether more junior faculty or trainees. Indeed, analyzing bibliometric trends including gender authorship trends, is more important given the importance of manuscript publications in successfully obtaining orthoapedic surgery residency positions. Data from the 2018 U S. Residency Match showed that successful applicants matching in orthopaedic surgery averaged 11.5 research experiences (abstracts, presentation, and publications) versus 6.7 for those that did not match in orthopaedic surgery [13].

New advances in technology have made it easier for multiple institutions to collaborate and contribute to work that is mutually beneficial to all parties involved [7,14,15]. Many researchers in the past guarded their work in an effort to deter added competition for funding, publications, and impactful discoveries. However, with improved speed and ease of communication, we posit that institutions with different resources and expertise have been encouraged to collaborate more than was previously possible. This observation, along with the increasing emphasis on publication for obtaining registrar/residency and/or faculty positions, leads us to anticipate that the number of co-authors and institutions per manuscript has increased over time for *JHS,* similar to other disciplines [[16], [17], [18]]. The primary purpose of this study was to analyze gender and authorship trends over the past 30 years for *JHSE,* to expand upon the previous *JHSA* study [2], and to compare volumes of a specialty journal published on different continents.

## 2 Materials and Methods

### 2.1 Data collection

Trends in authorship were analyzed over a 30 year period in the *JHSA* and *JHSE*. Data were collected in a manner similar to several other bibliometric analyses [17,[19], [20], [21], [22]]. Data were collected from one year of each of the past 4 decades (1985, 1995, 2005, and 2015). Publication data were downloaded into EndNote X7 (Thomson Reuters, New York, NY, 2013) for review and then transferred into a Microsoft Excel (Redmond, WA, 2013) file for further manipulation. The names of first and corresponding authors; corresponding author position (1st, 2nd, other, last); corresponding author country of origin (and state or province for those from the United States of America or Canada, respectively); number of institutions, countries, authors, printed pages, and number of references were added into the Excel file. Countries were grouped by regions. North America was defined as the United States of America and Canada; Mexico, Central America, and South America as Latin America; Europe as the European continent along with Russia and Turkey; and Asia as all Asian countries beginning east of Turkey, including the Middle East and Israel. The two other regions were Africa and Australia/New Zealand.

Manuscripts without author information were excluded, as were other publications that were not original research such as editorials, memorandums, commentaries, meeting notes, letters, and abstracts. Electronically published articles that were not printed until the following year were also excluded. The number of citations per article was analyzed as an estimation of research quality. Citation data was obtained from a Scopus search during July 2018. Since newer publications have less time to be cited, citation values were normalized by dividing total citations by its approximate age in years (2.5 for 2015, 12.5 for 2005, 22.5 for 1995, and 32.5 for 1985).

Author gender was identified for first and corresponding authors using the method described and validated by Mimouni et al. [20], and previously used by our group and others [2,17,21,22]. Briefly, the author's first name was entered into a Google-based website tool (http://www.gpeters.com/names/baby-names.php) that uses the Google database to determine whether a specific name is more strongly associated with the male or female gender. The program then provides a ratio quantifying that association. For example, the name “Alex” is 7.363 times more common in males than in females according to this program. Based on the validation studies, ratios of 3.0 and above were considered predictive of gender, and the predicted gender was assigned in our database. If the program produced a ratio less than 3.0, a Google search was conducted to find the author and confirm his or her gender. If that search was inconclusive, then the publication was excluded from gender analyses. For author gender combination studies (examining the gender of the first and corresponding authors on a manuscript), single author manuscript and manuscripts in which the first and corresponding author were the same person, were excluded from this analysis.

### 2.2 Statistical analysis

Continuous data are reported as the mean and standard deviation. Discrete data are reported as frequencies/percentages. Analyses between groups of continuous data were performed using non-parametric tests due to non-normal distribution of the data (Mann-Whitney U −2 groups; Kruskal-Wallis test −3 or more groups). Differences between groups of discrete data were analyzed by the Fisher's exact test (2 x 2 tables) and the Pearson's χ^2^test (greater than 2 x 2 tables). Trends over time for dichotomous categorical variables (2 x k tables) were assessed using the Cochran linear trend (CLT) test. A p < 0.05 was considered statistically significant for all tests.

## 3 Results

### 3.1 Journal of Hand Surgery (European volume)

A total of 450 manuscripts met inclusion criteria. Because of the low number of manuscripts from Africa (n = 9) and Latin America (n = 10), these regions were excluded from the regional analyses. There were significant increases over time (Table 1) in all bibliometric variables analyzed, except for the number of countries and in corresponding author gender. The only significant difference by region (Table 2) was in corresponding author position. There were differences based on first author gender for corresponding author position, number of institutions, normalized citations, references, and pages per manuscript (Table 3); all were greater for female first authors compared to male first authors. The number of references and pages were significantly higher for female corresponding authors (Table 3). The average percentage of female first authors was 13% and increased from 8.3% in 1985 to 19% in 2015 (P = 0.004, CLT) (Table 1). The average percentage of female corresponding authors was 8.7%, with no significant change over time (P = 0.096, CLT) (Table 1). Gender was identified for 439/450 manuscripts for *JHSE*. There were no significant differences for corresponding author gender by region (P > 0.05).

**Table 1:** Bibliometric variables by year for *Journal of Hand Surgery-European Volume (JHSE)*.

Variable	1985	1995	2005	2015	P-value
**Number of Papers**	75	167	107	101	
	Mean ± SD	Mean ± SD	Mean ± SD	Mean ± SD	
**Number of Authors**	2.3 (±1.1)	3.3 (±2.0)	3.7 (±1.7)	4.3 (±1.9)	<10^−6^
**Corresponding Author Position**	1.2 (±0.4)	1.3 (±0.7)	1.5 (±1.1)	2.0 (±1.6)	<10^−6^
**Number of Institutions**	1.1 (±0.4)	1.1 (±0.3)	1.2 (±0.5)	2.3 (±2.0)	0.00009
**Number of Countries**	1.1 (±0.2)	1.0 (±0.1)	1.1 (±0.3)	1.3 (±1.0)	0.11
**Number of Citations**	20.1 (±29.1)	23.5 (±21.6)	24.3 (±19.2)	9.5 (±9.2)	<10^−6^
**Number of Normalized Citations**	0.6 (±0.9)	1.1 (±1.0)	2.0 (±1.5)	3.8 (±3.7)	<10^−6^
**Number of References**	13.8 (±9.4)	17.9 (±16.1)	22.6 (±13.3)	23.5 (±10.6)	<10^−6^
**Number of Pages**	4.0 (±1.6)	4.8 (±2.5)	4.9 (±1.8)	7.3 (±2.7)	<10^−6^
**First Author Gender**					
**Female**	6	13	19	18	0.004
**Male**	66	152	87	78	
Corresponding Author Gender					
**Female**	6	8	13	11	.096
**Male**	67	158	93	83	

SD = Standard Deviation.

**Table 2:** Bibliometric variables by region for *Journal of Hand Surgery-European Volume (JHSE*).

Variable	North America	Europe	Asia	Australia/New Zealand	P-value
**Number of Papers**	81	266	76	19	
	Mean ± SD	Mean ± SD	Mean ± SD	Mean ± SD	
**Number of Authors**	3.3 (±1.4)	3.5 (±2.1)	3.6 (±1.7)	3.0 (±1.1)	0.31
**Corresponding Author Position**	1.9 (±1.3)	1.4 (±0.9)	1.5 (±1.1)	1.6 (±1.0)	0.00007
**Number of Institutions**	1.4 (±0.8)	1.4 (±1.2)	1.3 (±0.8)	1.6 (±1.9)	0.34
**Number of Countries**	1.1 (±0.3)	1.1 (±0.5)	1.2 (±0.7)	1.1 (±0.2)	0.29
**Number Citations**	23.3 (±22.2)	20.1 (±22.4)	17.3 (±17.6)	16.2 (±13.9)	0.43
**Number of Normalized Citations**	1.8 (±2.4)	1.7 (±2.1)	2.1 (±3.1)	1.7 (±2.3)	0.84
**Number of References**	20.2 (±12.4)	19.0 (±15.7)	18.1 (±11.6)	14.6 (±7.0)	0.25
**Number of Pages**	5.4 (±2.3)	5.2 (±2.7)	5.5 (±2.2)	4.6 (±1.5)	0.25
**Corresponding Author Gender**					
**Female**	4	29	0	5	0.14
**Male**	77	230	19	67	

SD = Standard Deviation.

**Table 3:** Bibliometric variables by gender for first and corresponding author for *Journal of Hand Surgery-European Volume (JHSE)*.

Variable	First Author			Corresponding Author		
	Female	Male	P	Female	Male	P-value
**Number of Papers**	56	383		38	401	
	Mean ± SD	Mean ± SD		Mean ± SD	Mean ± SD	
**Number of Authors**	3.7 (±1.8)	3.4 (±1.9)	0.2	3.6 (±1.6)	3.4 (±1.9)	0.36
**Corresponding Author Position**	1.8 (±1.6)	1.5 (±1.0)	0.04	1.4 (±1.0)	1.5 (±1.0)	0.11
**Number of Institutions**	1.7 (±1.5)	1.3 (±1.0)	0.03	1.7 (±1.4)	1.3 (±1.1)	0.20
**Number of Countries**	1.1 (±0.4)	1.1 (±0.5)	0.2	1.2 (±0.5)	1.1 (±0.5)	0.26
**Number of Citations**	21.0 (±22.3)	19.9 (±21.2)	0.8	24.1 (±25.1)	19.8 (±21.0)	0.28
**Number of Normalized Citations**	2.2 (±2.4)	1.7 (±2.3)	0.04	2.6 (±3.0)	1.70 (±2.2)	0.12
**Number of References**	25.6 (±23.9)	17.8 (±12.0)	0.02	26.4 (±22.1)	18.0 (±13.0)	0.012
**Number of Pages**	6.1 (±2.9)	5.1 (±2.4)	0.02	6.3 (±3.0)	5.1 (±2.4)	0.012

SD = Standard Deviation.

### 3.2 Journal of Hand Surgery (American volume)

There were 763 manuscripts which met inclusion criteria. Due to the low number of manuscripts from Africa (n = 7) and Latin America (n = 13), these regions were excluded from regional analyses. There were significant increases over time (Table 4) in all bibliometric variables analyzed. There were also significant differences by region (Table 5) for author number, corresponding author position, and number of citations. There were significant differences based on the first author gender for all variables except number of countries and normalized citations (Table 6). All significant variables were greater for female first authors as compared to male first authors, except for the number of citations. There were significant differences based on corresponding author gender for number of citations, normalized citations, references, and pages for each manuscript (Table 6). All significantly different variables were greater for female corresponding authors, except for the number of citations. The overall average percentage of female first authors was 14% and increased from 7.0% in 1985 to 24% in 2015 (P < 10^−6^, CLT) (Table 4). The overall average percentage of female corresponding authors was 9.7% and increased from 6.2% in 1985 to 18% in 2015 (P = 0.00002, CLT) (Table 4). Gender was identified for 756/763 manuscripts for *JHSA*. There were no significant differences observed based on corresponding author gender differences by region (P > 0.05).

**Table 4:** Bibliometric variables by publication year for *Journal of Hand Surgery-American Volume (JHSA)*.

Variable	1985	1995	2005	2015	P-value
**Number of Papers**	179	175	174	235	
	Mean ± SD	Mean ± SD	Mean ± SD	Mean ± SD	
**Number of Authors**	2.7 (±1.3)	3.2 (±1.3)	4.0 (±1.4)	4.7 (±1.6)	<10^−6^
**Corresponding Author Position**	1.3 (±0.7)	1.6 (±0.9)	2.0 (±1.5)	2.7 (±1.4)	0.0001
**Number of Institutions**	1.3 (±0.6)	1.3 (±0.6)	1.6 (±1.0)	1.4 (±0.8)	<10^−6^
**Number of Countries**	1.0 (±0.3)	1.1 (±0.2)	1.1 (±0.3)	1.1 (±0.3)	<10^−6^
**Number of Citations**	34.3 (±45.0)	37.5 (±34.8)	29.7 (±29.6)	4.9 (±4.9)	<10^−6^
**Number of Normalized citations**	1.1 (±1.4)	1.7 (±1.5)	2.4 (±2.4)	2.0 (±2.0)	<10^−6^
**Number of References**	13.8 (±9.4)	17.9 (±16.1)	22.6 (±13.3)	23.5 (±10.6)	<10^−6^
**Number of Pages**	5.2 (±2.5)	5.6 (±2.5)	6.5 (±2.1)	6.8 (±2.0)	<10^−6^
**First Author Gender**					
**Female**	12	16	22	55	<10^−6^
**Male**	160	157	150	171	
**Corresponding Author Gender**					
**Female**	11	9	11	41	0.000006
**Male**	162	164	162	184	

SD = Standard Deviation.

**Table 5:** Bibliometric variables by region for *Journal of Hand Surgery-American Volume (JHSA)*.

Variable	North America	Europe	Asia	Australia/New Zealand	P-value
**Number of Papers**	537	104	96	13	
	Mean ± SD	Mean ± SD	Mean ± SD	Mean ± SD	
**Number of Authors**	3.6 (±1.6)	3.9 (±1.5)	4.1 (±1.7)	4.2 (±2.4)	0.02
**Corresponding Author Position**	2.2 (±1.6)	1.3 (±0.9)	1.7 (±1.5)	1.7 (±1.4)	<10^−6^
**Number of Institutions**	1.4 (±0.8)	1.3 (±0.7)	1.4 (±0.7)	1.2 (±0.4)	0.28
**Number of Countries**	1.1 (±0.3)	1.1 (±0.5)	1.1 (±0.3)	1.1 (±0.3)	0.20
**Number of Citations**	27.7 (±36.4)	21.4 (±29.2)	16.0 (±20.3)	12.8 (±13.0)	0.00006
**Number of Normalized Citations**	1.9 (±2.0)	1.8 (±1.9)	1.4 (±1.4)	1.2 (±1.1)	0.12
**Number of References**	19.2 (±13.2)	22.0 (±12.4)	20.7 (±13.4)	18.5 (±10.4)	0.096
**Number of Pages**	6.1 (±2.4)	6.2 (±2.4)	6.1 (±2.1)	6.2 (±2.0)	0.90
**Corresponding Author Gender**					
**Female**	48	15	9	0	0.21
**Male**	489	88	82	13	

SD = Standard Deviation.

**Table 6:** Bibliometric variables by gender for first and corresponding author for *Journal of Hand Surgery-American Volume (JHSA)*.

Variable	First Author			Corresponding Author		
	Female	Male	P	Female	Male	P-value
**Number**	106	650		73	683	
	Mean ± SD	Mean ± SD		Mean ± SD	Mean ± SD	
**Number of Authors**	4.0 (±1.6)	3.6 (±1.6)	0.05	4.0 (±1.6)	3.7 (±1.6)	0.07
**Corresponding Author Position**	2.5 (±1.9)	1.9 (±1.5)	0.00002	1.9 (±1.5)	2.0 (±1.6)	0.52
**Number of Institutions**	1.5 (±0.7)	1.4 (±0.7)	0.05	1.4 (±0.7)	1.4 (±0.8)	0.65
**Number of Countries**	1.1 (±0.3)	1.1 (±0.3)	0.16	1.1 (±0.3)	1.1 (±0.3)	0.24
**Number of Citations**	21.9 (±3.5)	25.6 (±33.7)	0.005	20.5 (±29.7)	25.4 (±34.0)	0.03
**Number of Normalized Citations**	2.0 (±2.2)	1.7 (±1.9)	0.09	2.2 (±2.4)	1.7 (±1.9)	0.05
**Number of References**	22.2 (±11.8)	19.3 (±13.2)	0.005	22.4 (±11.8)	19.4 (±13.2)	0.02
**Number of Pages**	6.7 (±2.4)	6.0 (±2.3)	0.003	6.5 (±2.1)	6.0 (±2.3)	0.04

SD = Standard Deviation.

### 3.3 Differences between *JHSE* and *JHSA*

There were significant differences observed between the journals for author number, corresponding author position, numbers of institutions, references, and printed pages. All were higher for *JHSA* except for number of institutions (Table 7). With regard to institutions, although both *JHSE* and *JHSA* had an average of 1.4 institutions contributing to each manuscript the spread was larger for *JHSE* (1 to 12 versus 1 to 6), resulting in the statistically significant difference. As shown in Table 8, no significant differences were observed between the journals in the percentage of female first authors, female corresponding authors, or single author manuscripts. However, there were significant differences between the journals regarding region of manuscript origin (P < 10^−6^). There were no differences between journals in author gender combinations. *JHSA* had a more rapid increase in female authorship over time (Table 1, Table 4). For female first authors, *JHSA* increased 17% compared to an 11% increase for *JHSE*. For female corresponding authors, *JHSA* increased 12% compared to a 4.6% reduction for *JHSE* (Table 1, Table 4). While *JHSA* showed a steeper increase in female authorship over time, it should be noted that *JHSE* already had a higher percentage of female authorship beginning in 1985.

**Table 7:** Differences between *Journal of Hand Surgery-American Volume (JHSA)* and *Journal of Hand Surgery-European Volume (JHSE)* for continuous bibliometric variables.

Variable	*JHSA*	*JHSE*	P-value
**Number of Papers**	763	450	
	Mean ± SD	Mean ± SD	
**Number of Authors**	3.7 (±1.6)	3.4 (±1.9)	0.01
**Corresponding Author Position**	2.0 (±1.6)	1.5 (±1.1)	<10^−6^
**Number of Institutions**	1.4 (±0.8)	1.4 (±1.1)	0.0004
**Number of Countries**	1.1 (±0.3)	1.1 (±0.5)	0.76
**Number of Citations**	24.9 (±33.8)	20.0 (±21.3)	0.93
**Number of Normalized Citations**	1.8 (±1.9)	1.8 (±2.3)	0.13
**Number of References**	19.7 (±13.0)	18.8 (±14.2)	0.03
**Number of Pages**	6.1 (±2.3)	5.3 (±2.5)	<10^−6^

SD = Standard Deviation.

**Table 8:** Differences between *Journal of Hand Surgery-American Volume (JHSA)* and *Journal of Hand Surgery-European Volume (JHSE)* for categorical bibliometric variables.

Variable	*JHSA*	*JHSE*	% *JHSA*	% *JHSE*	P-value
Number of Papers	N	N	%	%	
**First Author**					
**Female**	106	56	14.0	12.8	0.60
**Male**	650	383	86.0	87.2	
**Corresponding Author**					
**Female**	73	38	9.7	8.7	0.61
**Male**	683	401	90.3	91.3	
**Single Author**					
**Yes**	52	43	6.8	9.6	0.10
**No**	711	406	93.2	90.4	
**Region**					
**Asia**	96	76	12.8	17.3	<10^−6^
**Australia/New Zealand**	13	19	1.7	4.3	
**Europe**	104	265	13.9	60.2	
**North America**	537	80	71.6	18.2	
**Author Gender Combination**^a^					
**FF**	10	2	3.4	1.7	0.64
**FM**	48	18	16.4	16.8	
**MF**	15	3	5.1	2.8	
**MM**	220	84	75.1	78.5	

a FF = both 1st and corresponding authors female, FM 1st author female and corresponding author male, MF = 1st author male and corresponding author female, and MM = both 1st and corresponding authors male.

## 4 Discussion

Over the past 30 year history of *JHSA* and *JHSE*, the average number of authors has increased. Authorship inflation is a well-known phenomenon [[16], [17], [18]]. This is not surprising considering the importance of publications to career advancement in academic medicine; thus, there may be more incentive for authors to include their name on as many publications as possible. A previous study demonstrated that some individuals accept unearned authorship and there may not be any penalty for authors who contribute nothing intellectually to a manuscript [23]. However, the rising number of authors may be a positive phenomenon and reflect efforts to increase collaboration [7,14,15]. Both journals saw the average number of institutions increase over time, especially *JHSE.* With advancements in technology, communication between institutions and countries is easier than ever before, increasing opportunities to collaborate*.* Additionally, researchers have access to manuscripts from journals around the globe, an impossible resource to imagine prior to the internet. The increasing number of references cited by each manuscript may be reflective of this advancement, as well as increasing collaboration, or this could possibly be due the ease of identifying other relevant studies due to increased search engine capabilities across many databases.

There are many advantages to collaboration, including potential increases in research productivity due to resource sharing and increased individual skill sets [5,6,24]. However, there can be drawbacks to collaboration between different countries, especially when one is more developed than the other [[15], [25]]. Such drawbacks are: equal opportunities for individual authors, competence of potential partners, respect between institutions, trust and confidence, and justice and fairness in collaboration.

Over the past few decades, there were many similar changes in the bibliometric variables for both *JHS* journals, including an increase in author number, corresponding author position, number of institutions, normalized citations, and pages per manuscript. When comparing the two journals, differences were observed in all variables except for the number of countries, citations, and normalized citations, which were similar. All values were higher for *JHSA*. These increases likely indicate more collaboration amongst the scientific community.

Female first and corresponding authors in *JHSE* and *JHSA* had a greater number of references and manuscript pages than male authors. Female first authors in *JSHE* and *JHSA* also had higher corresponding author positions (20% and 21%, respectively), more institutions (31% and 7.1%, respectively), and a greater number of normalized citations (30% and 20%, respectively). The latter may suggest that *JHS* female first authors are engaging in more impactful or more controversial research topics as their work is cited more often. It should be noted that for female corresponding authors the normalized citation number was also 53% and 28% higher, respectively for *JHSE* and *JHSA*. Although our study does not address the source of these differences, it is possible that female authors list more authors on their manuscripts due to increased willingness to collaborate with others as well as increased willingness to accredit those who contributed to the study. Taken together, it appears that for both journals, female first and/or corresponding authors are receiving more citations/year, indicating their work is receiving more acknowledgement from others in the field.

*JHSA* had a 3.5 times increase in female first authorship over time compared to 2.3 for *JHSE*. Only *JHSA* exhibited a significant change over time for female corresponding authors, although both journals showed an increase. Historically, first authors are often responsible for completion of the research and writing the manuscript [[26], [27], [28]], while corresponding authors are often the more senior researchers and are considered to have generated the research idea and supervised the research within their clinical division. As a result, these two positions are regarded as the most significant in terms of authorship credit and helpful for career advancement [7,^16^,^29^,30]. One possible explanation for the slower increase in female corresponding authorship could be that while the percentage of females entering medicine has increased, there has not been enough time for increased female population of senior positions in academic medicine. This is plausible for any field, but especially orthopaedic surgery as the gender gap has traditionally been so large [4,6,9]. As the percentages of both female first authors and corresponding authors have increased over the past 30 years, it is anticipated that these trends will continue to progress in the future as more women obtain faculty and academic medicine leadership positions.

From information gathered from the American Society for Surgery of the Hand about their membership in 2018, 17% of American hand surgeons were women while 20% of European hand surgeons were women (it should be noted that while primarily composed of orthopaedic surgeons, some hand surgeons are preliminarily trained in plastic surgery or general surgery). These data are encouraging, as they appear to represent an increase from previous figures. Indeed, the European Pharmaceutical Market Research Association Foundation Committee report in 2006 noted that on average, only 14% of orthopaedic surgeons worldwide were women [8]. This previous data did not include all countries, but it did include Canada, France, Germany, Italy, Japan, Spain, United Kingdom, and the United States. Of relevance to the present study, in 2015, 24% and 19% of first authors were female for *JHSA* and *JHSE*, respectively; while, 18% and 11% of corresponding authors were female for *JHSA* and *JHSE*, respectively. Thus, the gender composition of first authors and orthopaedic surgeons that are members of the American Society for Surgery of the Hand are similar. However, the gender composition of corresponding authors appears to be lower, especially for those originating from Europe. As the corresponding author is typically in the field longer than a first author (if the first author is not also the corresponding author), corresponding author data may lag behind the first author data, if they are a reflection of the percentage of women in the field.

As of 2018, the impact factor of *JHSE* was 2.648 and *JHSA* 1.776. Impact factor reflects the average number of times all manuscript in the journal were cited over the previous 2 years divided by how many items were published over that same 2 year period [24]. As an additional citation metric, we studied the number of times an article was cited normalized by the age of the manuscript. *JHSE* had a greater number of normalized citations, explaining the higher impact factor; however, *JHSE* had a significant increase in the number of citations for every decade, and *JHSA* an increase for every decade except for a slight drop in 2015. In general this indicates that the manuscripts in both journals are being cited more frequently. This could be due to easier access to manuscripts, and/or could also be due to the recent increased emphasis on the practice of evidence based medicine. Of note, for *JHSE*, female first authors were cited more often than their male counterparts (same trend but not statistically significant for *JHSA*); whereas for *JHSA*, female corresponding first authors were cited more often than their male counterparts (same trend but not statistically significant for *JHSE*). These intriguing findings may suggest that within the field of hand surgery, women are beginning to contribute more impactful research findings. This is likely due to the increase in female representation but may also reflect a benefit of their willingness to collaborate.

As with all studies, there were limitations. Although it would have been interesting, we were unable to identify a validated method to assess author ethnicity; therefore, the region of origin of the corresponding author could be used as the best proxy for this measurement. Additionally, in this study we examined one year per decade, rather than all manuscripts over time. While we understand that there could be changes within a decade which could potentially impact the data (such as economic fluxes), we believe this unlikely for a couple of reasons. For example, we previously validated the methodology of a 10% random sampling of manuscripts from a journal; there were no significant differences when compared to the decade procedure as used here [22]. Moreover, from inception to acceptance of a publication, each study may have markedly different timelines depending on a variety of factors. Thus, economic fluxes in one year would likely make a minimal impact on manuscripts published in that year or the following year. As mentioned above, the accuracy of our gender-based analyses are dependent on the accuracy of the website-based tool and the 3.0 ratio cut-off. However, this technique and website have been previously validated [20], and multiple investigators have published findings using this tool [2,17,21,22]. Finally, there are many other journals that could have been studied; however, we selected two well respected journals focused on hand surgery. Examination of additional journals would have required considerably more effort due to the labor intensive nature of manually collecting this data. This was unfortunately not feasible with our limited resources and time.

### 4.1 Conclusions

This study analyzed bibliometric trends in the orthopaedic hand surgery field by examining trends in *JHSA* and *JHSE* over the past 30 years. Collaboration between institutions and countries increased over time for both volumes, likely due to the increasing ease of sharing information. Regarding author gender, both journals showed an increase in female first and corresponding authorship over time, and these were statistically significant for first author in both volumes and corresponding author in *JHSA*. Filardo et al. (2016) showed that female first authorship in medical journals has increased over the past 20 years but has recently plateaued and even declined in some journals [4]. *JHSA* and *JHSE* do not demonstrate this plateau and demonstrate continued increases in the percentages of female authors (both first and corresponding). As more females enter the field of orthopaedic surgery in general, and hand surgery in particular, this trend should continue to improve in the future.

## Provenance and peer review

Not commissioned, externally peer reviewed.

## Funding statement

This work was supported in part by the Department of Orthopaedic Surgery, 10.13039/100010249Indiana University School of Medicine (10.13039/501100009914MAK, RTL), the Garceau Professorship Endowment and Rapp Pediatric Orthopaedic Research Fund, Riley Children's Foundation (RTL), and the Ruth Lilly Medical Library (ECW). This work was also supported by the Ralph W. and Grace M. Showalter Research Trust (MAK).

## Details of informed consent

N/A.

## Level of evidence

Not Applicable.

## Ethical approval

Not required.

## Funding

Internal funding only for faculty time/effort.

## Author contribution

AWP: investigation, data curation, writing original draft, visualization, writing review and editing. MKS: investigation, data curation, writing original draft, visualization, writing review and editing. ZG: investigation, data curation, writing review and editing. GA: investigation, data curation, writing review and editing. AM: investigation, data curation, writing review and editing. EW: conceptualization, methodology, validation, resources, writing review and editing, supervision, project administration, funding acquisition. RTL: conceptualization, methodology, validation, resources, writing review and editing, supervision, project administration, funding acquisition. MAK: conceptualization, methodology, validation, resources, writing review and editing, supervision, project administration, funding acquisition.

## Trial registry number

Name of the registry: N/A.

Unique Identifying number or registration ID: N/A.

Hyperlink to the registration (must be publicly accessible): N/A.

## Guarantor

Melissa Kacena, Randall Loder, and Elizabeth Whipple accept full responsibility for the work.

## Declaration of competing interest

The authors declared no potential conflicts of interest with respect to the research, authorship, and/or publication of this article.
